# Adenosine A_3_ receptor as a novel therapeutic target to reduce secondary events and improve neurocognitive functions following traumatic brain injury

**DOI:** 10.1186/s12974-020-02009-7

**Published:** 2020-11-12

**Authors:** Susan A. Farr, Salvatore Cuzzocrea, Emanuela Esposito, Michela Campolo, Michael L. Niehoff, Timothy M. Doyle, Daniela Salvemini

**Affiliations:** 1 Veterans Affairs Medical Center, 915 N Grand Blvd, St. Louis, MO 63106 USA; 2 Department of Internal Medicine, Division of Geriatric Medicine, Saint Louis University School of Medicine, 1402 S. Grand Blvd, St. Louis, MO 63104 USA; 3 Department of Pharmacology and Physiology, Saint Louis University School of Medicine, 1402 S. Grand Blvd, St. Louis, MO 63104 USA; 4 Henry and Amelia Nasrallah Center for Neuroscience, Saint Louis University School of Medicine, 1402 S. Grand Blvd, St. Louis, MO 63104 USA; 5 Department of Clinical and Experimental Medicine and Pharmacology, University of Messina, 98122 Messina, Italy

**Keywords:** Traumatic brain injury, A_3_AR, Neuroinflammation, Cognitive impairment, NLRP3

## Abstract

**Background:**

Traumatic brain injury (TBI) is a common pathological condition that presently lacks a specific pharmacological treatment. Adenosine levels rise following TBI, which is thought to be neuroprotective against secondary brain injury. Evidence from stroke and inflammatory disease models suggests that adenosine signaling through the G protein-coupled A_3_ adenosine receptor (A_3_AR) can provide antiinflammatory and neuroprotective effects. However, the role of A_3_AR in TBI has not been investigated.

**Methods:**

Using the selective A_3_AR agonist, MRS5980, we evaluated the effects of A_3_AR activation on the pathological outcomes and cognitive function in CD1 male mouse models of TBI.

**Results:**

When measured 24 h after controlled cortical impact (CCI) TBI, male mice treated with intraperitoneal injections of MRS5980 (1 mg/kg) had reduced secondary tissue injury and brain infarction than vehicle-treated mice with TBI. These effects were associated with attenuated neuroinflammation marked by reduced activation of nuclear factor of kappa light polypeptide gene enhancer in B cells (NFκB) and MAPK (p38 and extracellular signal-regulated kinase (ERK)) pathways and downstream NOD-like receptor pyrin domain-containing 3 inflammasome activation. MRS5980 also attenuated TBI-induced CD4^+^ and CD8^+^ T cell influx. Moreover, when measured 4–5 weeks after closed head weight-drop TBI, male mice treated with MRS5980 (1 mg/kg) performed significantly better in novel object-placement retention tests (NOPRT) and T maze trials than untreated mice with TBI without altered locomotor activity or increased anxiety.

**Conclusion:**

Our results provide support for the beneficial effects of small molecule A_3_AR agonists to mitigate secondary tissue injury and cognitive impairment following TBI.

## Introduction

Traumatic brain injury (TBI) is a common pathological condition presently lacking a specific pharmacological treatment approved by the Food and Drug Administration [1]. TBI results in long-term physical and cognitive deficits arising from primary and secondary injuries. The primary injury starts at the moment of TBI impact and is characterized by the disruption of blood brain barrier and blood vessels that contribute to the formation of brain edema [2, 3]. This triggers a secondary injury cascade that includes the activation of brain-resident astrocytes and microglia and enrollment of peripheral immune cells into the brain. As a result, these immune components may promote cell death during the early phase after TBI impact and contribute to subsequent neurological impairments during the later stages [2, 4–6]. Therefore, targeting secondary events following TBI could be a promising strategy for the development of novel therapy [7].

Adenosine levels rise in the cerebrospinal fluid and interstitial space 1 h after TBI in animals and humans [8, 9]. Further adenosine release occurs during periods of mismatched cerebral blood flow and cerebral metabolic rate of oxygen consumption in TBI patients [10]. Upon release, adenosine can bind to one of four G protein-coupled receptor (GPCR) subtypes: A_1_AR, A_2A_AR, A_2B_AR, and A_3_AR [11].

Our knowledge of the role of adenosine signaling during TBI has been limited to its action at A_1_AR and A_2A_AR. In TBI models, A_1_AR signaling has been shown to be neuroprotective [12] and reduce the severity of secondary injury [13]. In contrast, A_2A_AR activation is detrimental and increases TBI-induced cognitive impairment [14]. A_2A_AR inhibition was found to provide antiinflammatory and neuroprotective effects that corresponded with improved histopathological outcomes over the course of the study [15] and antagonism of A_2A_AR signaling with caffeine after TBI reduced secondary brain injury and improved cognitive function [16, 17]. Collectively, these data point to a beneficial role for adenosine signaling during TBI and suggest adenosine signaling may be a good pharmacological target for treating TBI. However, targeting A_1_AR and A_2A_AR as a therapeutic approach to reduc secondary injury and loss of cognitive function following TBI have been limited. Systemic administration of A_1_AR agonists can produce serious adverse cardiovascular effects [18]. The use of caffeine has been linked to increased acute granulocytosis, edema, and disruption of the blood–brain barrier in animals [17] and chronic administration can reduce the recovery of motor function in patients [16].

The role of A_3_AR in TBI is not known. A_3_AR signaling has demonstrated antiinflammatory effects in models of autoimmune inflammatory diseases and chronic neuropathic pain [19, 20] as well as anticancer and cardioprotective properties [21]. Accordingly, orally bioavailable small molecule, receptor subtype-selective A_3_AR agonists, IB-MECA (CF101, picladenoson) and Cl-IB-MECA (CF102, namodenosin) have been developed and advanced to Phase II/III for autoimmune inflammatory conditions and cancer with good safety profiles [22–24]. In the brain, A_3_AR is generally expressed at low levels [11, 21, 25], yet immunohistochemical and radioligand binding studies have demonstrated A_3_AR expression clusters in various brain regions, including the hippocampus and cortex [26]. Studies in animal stroke models found improved ischemic outcome by reducing infarct volume and inflammatory cell infiltration with A_3_AR agonist treatment [27, 28]. In contrast, A_3_AR^−/−^ knockout mice were found to be more susceptible to hypoxia-induced hippocampal nerve death and exhibited increased cognitive decline than wild-type mice [29]. Given the apparent anti-neuroinflammatory and neuroprotective effects associated with A_3_AR signaling in the brain, we investigated whether A_3_AR activation improves pathological outcomes of secondary injury by attenuating inflammation and preserves cognitive function in animal models of TBI. Here, we used the highly-selective A_3_AR agonist MRS5980 (> 10,000 fold selectivity         versus A_1_AR or A_2A_AR) [30, 31] that has been reported to only bind the A_3_AR in a full GPCRome and kinome screen [32] and whose effects are fully blocked by the A_3_AR antagonist MRS1523 [33].

## Materials and methods

### Materials

MRS5980 ((1*S*,2*R*,3*S*,4*R*,5*S*)-4-(2-((5-chlorothiophen-2-yl)ethynyl)-6-(methylamino)-9*H*-purin-9-yl)-2,3-dihydroxy-*N*-methylbicyclo[3.1.0]hexane-1-carboxamide, gift of Kenneth A. Jacobson, NIDDK, National Institutes of Health) was prepared as described recently [34]. All other chemicals were purchased from the highest commercial grade available. All stock solutions were prepared in non-pyrogenic saline (0.9% NaCl, Baxter, Milan, Italy) or dimethyl sulfoxide (Sigma-Aldrich, St. Louis, MO, USA).

### Experiment animals

Male CD1 mice were used at 10 and 12 weeks of age (25 to 30 g) from Envigo (Italy) for controlled cortical impact TBI studies (Study 1) or from Charles River (Wilmington, MA) for closed-head weight drop TBI studies (Study 2). Mice were housed in individual cages (five per cage) and maintained under a 12:12 hour light/dark cycle at 21 ± 1 °C and 50 ± 5% humidity. Regular laboratory diet (Study 1) or PMI Nutrition LabDiet 5001 (Study 2) and tap water were available ad libitum.

### Study design

All animals were sex-, age- and weight-matched and experimentally naïve prior to head injury. To assure reproducibility, data were compiled from two to three experiments with equal number of animals in each group and behavioral experiments and corresponding biochemical assays were started and performed on different days with experimenters blinded to treatment conditions.

### Study 1: controlled cortical impact traumatic brain injury

Mice were randomly allocated into the following groups: (1) sham: mice were subjected to equal surgical procedures except for TBI and were kept under anesthesia for the duration of the surgery (*n* = 25); (2) TBI: mice were subjected to brain injury then administered an intraperitoneal (i.p.) injection of vehicle (saline at 5% dimethyl sulfoxide (DMSO)) 1 h and 4 h after trauma (*n* = 25) and (3) TBI + MRS5980: mice were subjected to brain injury then administered an i.p. injection of MRS5980 (1 mg/kg, i.p.) 1 h and 4 h after trauma (*n* = 25).

Traumatic brain injury was induced in mice by a controlled cortical impactor (CCI) as previously described [35, 36]. Briefly, a craniotomy of the right hemisphere encompassing the bregma and lambda between the sagittal suture and the coronal ridge was performed with a micro motor hand piece and drill (UGO Basile SRL, Comerio Varese, Italy). The resulting bone flap was removed and the cranial aperture was enlarged with cranial rongeurs (New Adalat Garh, Roras Road, Pakistan). A cortical contusion was made using the controlled stereotaxic impactor (Leica, Milan, Italy) on the exposed cortex (tip diameter: 4 mm; cortical contusion depth: 3 mm; impact velocity: 1.5 m/s). This generates brain injury of moderate severity [37]. Immediately after injury, the skin incision was secure with nylon sutures, and 2% lidocaine jelly in the lesion was used to reduce pain. No seizures or deaths were observed in any of these mice. All groups were sacrificed 24 h post-injury for histopathological and biochemical analyses.

### Study 2: closed head weight-drop traumatic brain injury

Mice were randomly assigned to the following groups: (1) sham: mice were anesthetized with isoflurane and placed in the apparatus but not subjected to head injury. Mice were administered i.p. injections of vehicle (saline with 5% DMSO) after 1 h every 2 days for the duration of the study (*n* = 11); (2) TBI + vehicle: mice were anesthetized with isoflurane and place in the apparatus and subjected brain injury. Mice were administered i.p. injections of vehicle after 1 h and every 2 days for the duration of the study (*n* = 11); and (3) TBI + MRS5980: mice were anesthetized with isoflurane and place in the apparatus and subjected to head injury. Mice were administered i.p. injections of MRS5980 (1 mg/kg) after 1 h and every 2 days for the duration of the study (*n* = 11).

Traumatic brain injury was induced in mice using the closed-head concussive method [38]. Briefly, mice were anesthetized with 2–4% isoflurane (confirmed by the loss of corneal reflex and toe pinch reflex). Then, the animal’s head was placed an immobilization sponge board and positioned under a device consisting of a Plexiglas tube (inner diameter 13 mm) placed vertically over the animal’s head. A 30-g acrylic weight was dropped down the Plexiglas tube from an 80 cm height, striking the head in area encompassing right of the central suture, behind bregma and in front of lambda on the parietal lobe. Following this procedure, the animal’s respiration, heart rate and righting reflex were monitored to ensure recovery. Mice with abnormal reflexes or exhibited abnormal ambulation 1 h after trauma were eliminated from study and euthanized by CO_2_ asphyxiation. No signs of seizures were observed in any of the mice. Materials for the apparatus were made by Interstate Plastics, Incorporated, Sacramento, CA.

### Histopathological quantification of brain injury

The mouse brains were harvested 24 h after CCI, fixed in 10% (w/v) buffered formaldehyde and paraffin-embedded. Coronal sections (7 μm) from the perilesional brain area of each animal were deparaffinized with a decreasing concentrations of xylene and alcohol, then stained with hematoxylin and eosin. All sections were analyzed by using an Axiovision Zeiss microscope (Milan, Italy). Histopathological changes of the gray matter were blindly scored using a 5-point scale: 0, no lesion observed; 1, gray matter contained 1 to 5 eosinophilic neurons; 2, gray matter contained 5 to 10 eosinophilic neurons; 3, gray matter contained more than 10 eosinophilic neurons; 4, small infarction—less than one third of the gray matter area; 5, large infarction—more than half of the gray matter area [39, 40]. The scores from all the brain sections were averaged for a final score for an individual mouse.

### Quantification of infarct volume

The mouse brains were harvested 24 h after CCI and cut into 5 coronal slices of 2 mm thickness by using a McIlwain tissue chopper (Campdem instruments LTD). Slices were incubated in 2,3,5-triphenyltetrazolium chloride (TTC; 2%) at 37°C for 30 min and immersion fixed in 10% buffered formalin solution. Infracted area and volume were calculated as previously described [41] using digital images (Canon 4X, Canon Inc., China) and ImageJ software [42]. To account for brain edema, the infarct areas were corrected by subtracting the area of the contralateral hemisphere area from the ipsilateral hemisphere [43]. The corrected total infarct volume was estimated by summing the infarct area in every slice and multiplying it by slice thickness (2 mm).

### Western blot analysis

Cytosolic and nuclear extracts were prepared from fresh frozen brain sections 24 h after CCI as previously described [35]. The expressions of cleaved caspase 1 p20, NRLP3, IkBα, p-p38 and p-ERK 1/2 was quantitated using cytosolic fractions while NFκB was detected in nuclear fraction. The Western blot membranes were probed with antibodies for nuclear factor of kappa light polypeptide gene enhancer in B cells inhibitor, alpha (IkBα; 1:500; Santa Cruz Biotechnology), nuclear factor of kappa light polypeptide gene enhancer in B cells (NFκB; 1:1000; BD Transduction Laboratories), p-p38 (1:500; Santa Cruz Biotechnology; sc-17852-R), total p38 (1:500; SantaCruz Biotechnology, Santa Cruz CA, USA), phophorylated extracellular signal-regulated kinases (p-ERK 1/2; 1:500; Santa Cruz Biotechnology), total ERK 1/2 (1:500; Santa Cruz Biotechnology), cleaved caspase 1 p20 (1:500; Santa Cruz Biotechnology; sc-1597) or NRLP3 (1:500; Santa Cruz Biotechnology; sc-66846) at 4°C overnight in 1× phosphate-buffered saline (PBS), 5% (w/v), non-fat dried milk and 0.1% Tween-20. Membranes were incubated with peroxidase-conjugated bovine anti-rabbit IgG secondary antibody or peroxidase-conjugated goat anti-mouse IgG (1:2000; Jackson ImmunoResearch, West Grove, PA, USA) for 1 h at room temperature. Equal protein loading was assessed by incubated the blots in the presence of antibodies against glyceraldehyde 3-phosphate dehydrogenase (GAPDH; 1:5000; Santa Cruz Biotechnology) for cytosolic proteins and lamin A/C (1:1000; Santa Cruz Biotechnology) for nuclear proteins. The signals were visualized with enhanced chemiluminescence detection system reagent according to the manufacturer’s instructions (Super Signal West Pico Chemiluminescent Substrate, Pierce Thermo Scientific, Rockford, IL, USA). Relative expression of bands were calculated by densitometry using Bio-Rad ChemiDoc™ XRS + software and Image Quant TL, v2003. The expression levels of target proteins were standardized to GAPDH and/or Lamin A/C levels. Phosphorylated proteins were standardized to their total protein values.

### Immunofluorescence staining

Tissue segments containing the lesion (1 cm on each side of the lesion) were fixed in 10% (w/v) buffered formaldehyde 24 h after CCI and paraffin embedded as previously described [36]. After deparaffinization and rehydration, the tissue was boiled in 0.1 M citrate buffer for 1 min and blocked in 2% (v/v) normal goat serum in PBS for 20 min. Sections were incubated with mouse monoclonal with polyclonal mouse anti-CD4 (1:100, v/v, Santa Cruz Biotechnology, Dallas, TX, USA) or anti-CD8 (1:100, Santa Cruz Biotechnology, Dallas, TX, USA) antibodies in a humidified chamber overnight at 37 °C. Sections were washed with 1× PBS and incubated with secondary antibody FITC-conjugated anti-mouse Alexa Fluor-488 antibody (1:2000 v/v Molecular Probes, UK) for 3 h at 37 °C. Sections were washed and nuclei were stained with 2 μg/ml 4′ 6-diamidino-2-phenylindole (DAPI; Hoechst, Frankfurt, Germany) in 1× PBS. Sections were observed and photographed at × 100 magnification using a Leica DM2000 microscope (Leica). All images were digitalized at a resolution of 8 bits into an array of 2560 × 1920 pixels. Optical sections of fluorescence specimens were obtained using a helium-neon laser (543 nm), a UV laser (361–365 nm) and an argon laser (458 nm) at a 1-min, 2-s scanning speed with up to 8 averages; 1.5 μm sections were obtained using a pinhole of 250. Contrast and brightness were established by examining the most brightly labeled pixels and applying settings that allowed clear visualization of structural details while keeping the highest pixel intensities close to 200. The same settings were used for all images obtained from the other samples that had been processed in parallel. Digital images were cropped and figure montages prepared using Adobe Photoshop CS6 (Adobe Systems; Palo Alto, CA, USA).

### Cognitive behavioral tests

All mice in Study 2 were tested 1 week and again at 4 weeks after sham or closed head weight-drop traumatic brain injury.

#### Novel Object Recognition Place recognition Test (NORPT)

Novel object-place recognition test (NORPT) is a memory task that involves the hippocampus where the animal is tested on whether it the retains the memory of an object it was exposed to 24 h prior to testing [44]. This test exploits the tendency of mice to spend more time exploring new, novel objects than familiar objects. Thus, the greater the retention/memory of the familar object, the more time they will spend with the new object.

NORPT trials for mice 1 week after trauma began for 3 days prior to test day where they were allowed to habituate to the test arena (25 cm (length) × 25 cm (width) × 50 cm (height) for 5 min/day. On day 4, two identical objects were placed in the arena and the mouse was permitted to freely explore the arena for 5 min. The mouse was returned to its home-cage and the arena and the two objects were cleaned with 70% ethanol. The mouse was returned to the open arena 24 h later and one object was replaced by a novel object with different shape in a different location. The mice were allowed to explore for another 5 min. The familiar and novel objects were of the same material to avoid potential interference of deficits in sense of touch or smell. All sessions were recorded for later analysis. Sniffing, climbing, and touching the objects were regarded as the exploration behavior and exploration times of the familiar and novel object were scored manually by a trained technician blinded to treatment and were validated by a second investigator.

NORPT trials for mice 4 weeks after trauma began the day before test day where they were allowed to explore the two like objects for 5 min. The next day, they were placed back in the arena with one object from the day prior and one new object. The objects for these test session were made of different material and different shapes than the test at 1 week post-injury.

Mice that did not explore both objects were not included in the analysis. The discrimination index was calculated as (time with novel-time with familiar object)/total exploration time of both objects. The NOPRT was performed during the light phase of the day/night cycle.

#### T-Maze training and testing procedures

The T-maze is a complex memory task involving the hippocampus. Permanent and temporary lesions to 30% of the anterior portion of the hippocampus result in impaired learning and memory during the T maze trials [45]. The T-maze consisted of a black plastic alley with a start box at one end and two goal boxes at the other. The start box was separated from the alley by a plastic guillotine door that prevented movement down the alley until raised at the onset of training. An electrifiable floor of stainless steel rods ran throughout the maze to deliver a mild scrambled foot-shock.

Mice were tested in T-maze starting 4 weeks post-TBI. Mice were not permitted to explore the maze prior to training. A block of training trials began when a mouse was placed into the start box. The guillotine door was raised and a cue buzzer (door-bell type sounded at 55 dB) sounded simultaneously; 5 s later foot-shock (0.35 mA; Coulbourn Instruments scrambled grid floor shocker model E13-08) was applied. The arm of the maze the mouse entered on the first trial was designated “incorrect” and the mild foot-shock was continued until the mouse entered the other goal box, which in all subsequent trials was designated as “correct” for the particular mouse. At the end of each trial, the mouse was returned to its home cage until the next trial (inter-trial interval = 35 s). Retention was tested 1 week later by continuing training until mice reached the criterion of 5 avoidances in 6 consecutive trials. The results were reported as the number of trials to criterion for the retention test.

#### Open-field test

To determine if coginitive test performance was affected by changes in animal activity or anxiety due to trauma or MRS5890 treatment, the mice were placed in an open field 4 weeks after closed head weight-drop traumatic brain injury. The mice were allowed to freely roam an empty circular apparatus (67.5 cm) for 15 min and distance traveled in cm as well as the time spent in the center portion of the open field was recorded on an ANY-maze (San Diego Instruments, CA, USA).

#### Elevated plus maze test

To further assess potential alterations in the levels of activity and anxiety in our mice, we measured their performance in the elevated plus maze during week 4 after closed head weight-drop traumatic brain injury. The apparatus consists of 4 arms perpendicular to each other in the shape of a plus sign, elevated 50 cm above the floor. Each arm is 35.5 cm in length; two opposite arms are open while the other two opposite arms are enclosed, as previously described [38]. The mouse is placed in the central platform facing an enclosed arm and allowing it to freely explore the maze for 5 min. The number of entries into the open and closed arms and the time spent in open arms was recorded by the ANY-maze. Anxiety and activty was indicated by decreased time spent and the number of entries in the open arms. The test arena was wiped with a damp cloth after each trial.

#### Statistical analysis

All values in the figures and text are expressed as mean ± standard error of the mean (SEM) of N number of animals. In those experiments involving histology or immunohistochemistry, the pictures exhibited are representative of at least three experiments performed on different days. Data that did not pass Shapiro-Wilk normality testing were analyzed by Kruskal-Wallis with Dunn’s comparison. All other data were analyzed two-tailed, two-way ANOVA with Bonferroni comparisons or one-way ANOVA followed by a Dunnett’s comparisons. A *p* value < 0.05 was considered significant.

## Results

### MRS5980 attenuates gross histopathological changes to cortical tissue following CCI

**Fig. 1.**
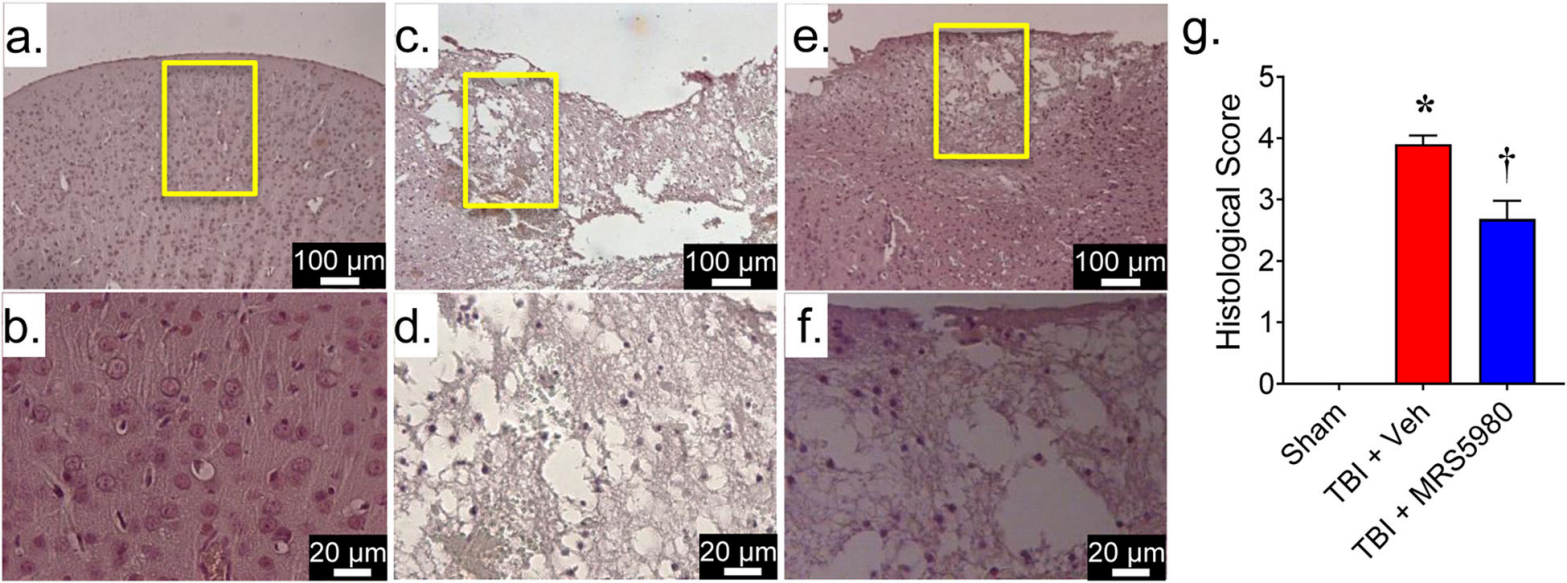
MRS5980 attenuates gross histopathological changes to cortical tissue following CCI TBI. **a**–**f** When compared to the intact brain structure of sham mice (**a** and higher magnification in **b**), tissue disorganization and inflammation increased in the penumbra area of the mouse brain sections 24 h after CCI TBI (TBI + Veh; **c** and higher magnification in **d**). These histopathological changes were attenuated in mice treated with MRS5980 (1 mg/kg, i.p.) after CCI TBI (**e** and higher magnification in **f**). **g** Histological scores of sagittal brain slices (three slices per animal). ND = not detectable; *yellow* box indicates region of higher magnification. Data are mean ± SEM of 10 mice/group and analyzed by Kruskal-Wallis [H(2)- = 24.17, *p* = 5.63 × 10^−6^] and Wilcoxon signed-rank test. *p < 0.05 vs. sham and **†***p* < 0.05 vs. TBI + Veh

**Fig. 2:**
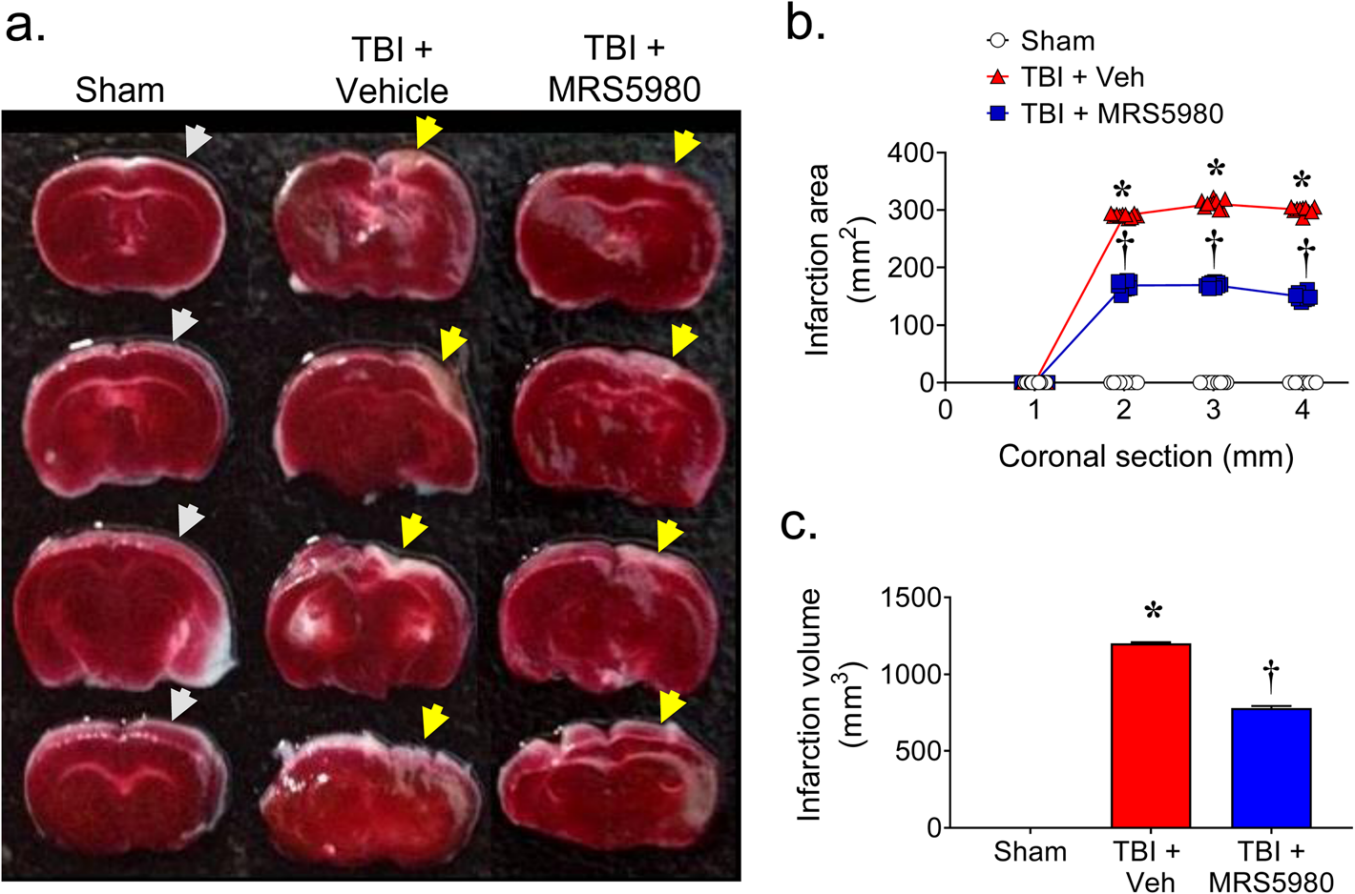
MRS5980 reduces the level of brain infarction following CCI TBI. **a** In serial coronal brain sections (2 mm thick) from sham group (side of sham surgery indicated by gray arrows), the level of TTC staining (red) was strong and uniformed indicating high metabolic and adequately perfused mouse brain. In contrast, the level of TTC staining in the brains from mice with TBI (side of CCI indicated by yellow arrows) and treated with vehicle (24 h after CCI TBI) was reduced overall and non-uniform, indicating increased brain infarction (white unstained regions). In brains from mice with CCI TBI (side of CCI indicated by yellow arrow) and treated with MRS5980 (1 mg/kg; i.p.), the level of TTC staining was greater and the regions of infarction (white unstained regions) were less than in untreated mice with TBI. **b**, **c** Morphometric analyses of infarct area and volume in TTC-stained brain slices. ND = not detectable. Data are individual value (**b**) or mean ± SEM (**c**) of 10 mice/group and analyzed by two-tailed, (**b**) two-way ANOVA [*F*(6, 81) = 2908; *p* = 2.32 × 10^−92^, η^2^_p_ = 0.995] with Bonferroni comparisons or (**c**) one-way ANOVA [*F*(2, 27) = 5006; *p* = 2.00 × 10^−35^, η^2^ = 0.997] with Dunnett’s comparisons. *p < 0.05 vs. sham and **†***p* < 0.05 vs. TBI + Veh

Consistent with previous reports [35, 36, 46, 47], the examination of brain sections from mice 24 h after sham injury (Fig. 1a, b, g) or CCI-induced TBI (Fig. 1c, d, g) revealed increased tissue disorganization and white matter alteration in the brain parenchyma of the perilesional area of mice with TBI. Moreover, the degree of brain infarction and necrotic tissue was greater in mice with TBI mice than sham mice (Fig. 2). However, mice administered MRS5980 (1 mg/kg; i.p) at 1 h and 4 h after TBI had significantly less brain tissue damage (Fig. 1e, f, g) and lesion volume (Fig. 2) when examined 24 h after trauma.

### MRS5980 attenuates the activation of NFκB and MAPKs pathways following CCI

**Fig. 3:**
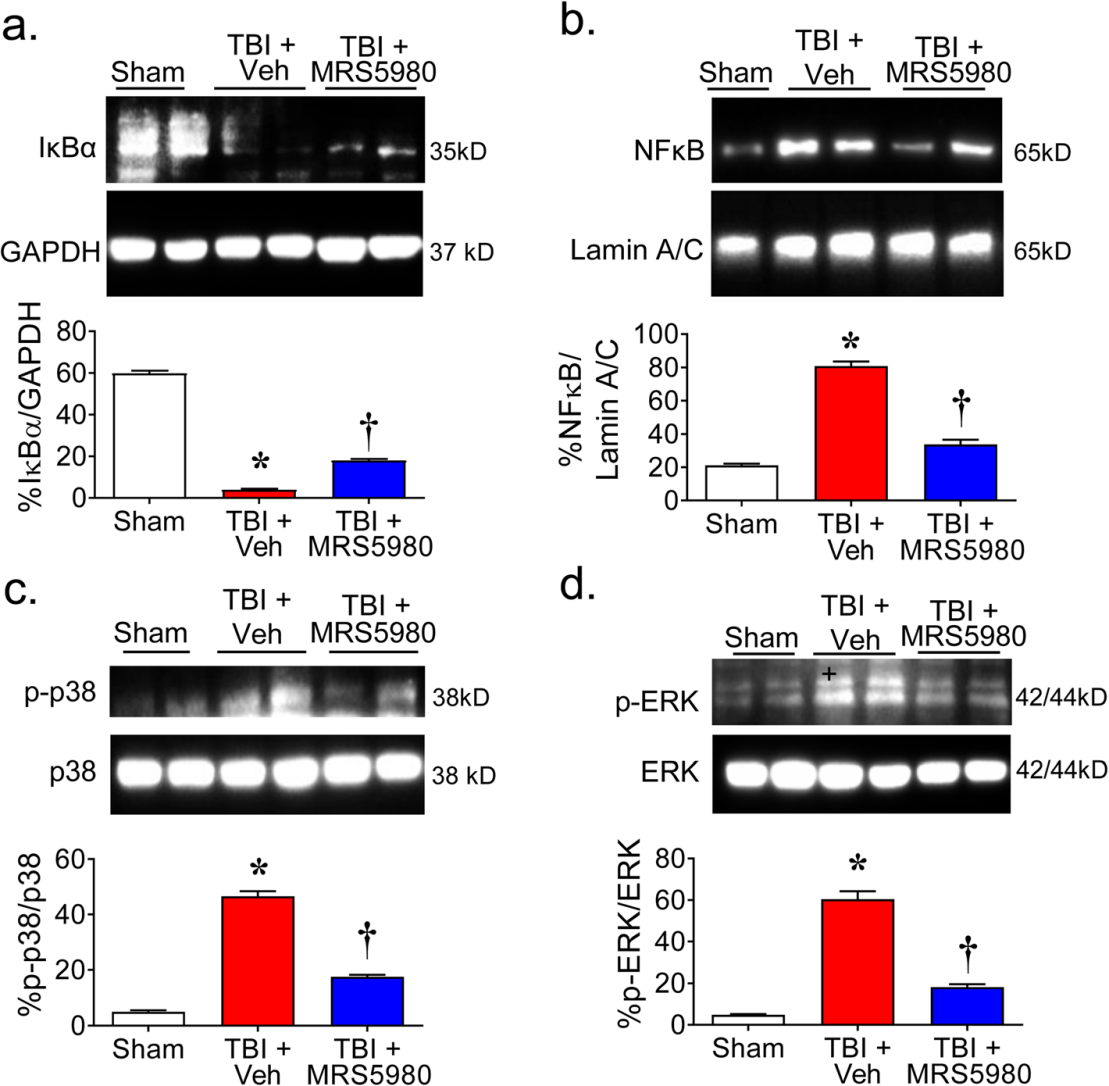
MRS5980 attenuates the activation of NFκB and MAPKs pathways following CCI TBI. When compared to sham mice, the levels of cytoplasmic IκBα were decreased (**a**) and the levels of nuclear NFκB p65 (**b**) and phosphorylated p38 (**c**) and ERK1/2 (**d**) increased in brain tissue from mice 24 h after CCI TBI that were treated with vehicle. These events were attenuated in mice with CCI TBI and treated with MRS5980 (1 mg/kg; i.p.; **a**–**d**). Uncropped blot images are shown in the supplementary information (Figures S1, S2, S3 and S4). Data are mean ± SEM for 5 mice/group and analyzed by two-tailed, one-way ANOVA with Dunnett’s comparisons [**a** *F*(2, 12) = 1098, *p* = 2.57 × 10^−14^, η^2^ = 0.995; **b** *F*(2, 12) = 180.0, *p* = 1.13 × 10^−9^, η^2^ = 0.968; **c** *F*(2, 12) = 325.5, *p* = 3.52 × 10^−11^; η^2^ = 0.992, **d** *F*(2, 12) = 325.5, *p* = 3.52 × 10^−11^, η^2^ = 0.992]. *p < 0.05 vs. sham and †*p* < 0.05 vs. TBI + Veh

NFκB [48–50] and MAPK (p38 and ERK) [50–52] signaling pathways have been shown to be activated in perilesional brain tissue in animal models of CCI TBI. Consistent with these reports, we found that TBI resulted in reduced cytoplasmic expression of the endogenous NFκB inhibitor IκBα (Fig. 3a) and increased nuclear translocation of NFκB p65 (Fig. 3b) in the perilesional brain tissue 24 h after injury, indicating the activation of the NFκB pathway. Likewise, we found phosphorylation of p38 (Fig. 3c) and ERK (Fig. 3d) increased following CCI TBI, indicating similar activation of MAPK signaling.

As part of their antiinflammatory mechanism of action, A_3_AR agonists have been shown to attenuate NFκB and MAPK signaling in number of autoimmune inflammatory disease models [31, 53]. When mice were treated with MRS5980 (1 mg/kg; i.p) 1 h and 4 h after TBI, we found increased cytoplasmic IκBα (Fig. 3a) and reduced nuclear NFκB p65 (Fig. 3b) and phosphorylation of p38 (Fig. 3c) and ERK (Fig. 3d) in the perilesional brain tissue 24 h after injury when compared to mice with TBI and treated with vehicle.

### MRS5980 attenuates NLRP3-inflammasome following CCI

Inflammatory NFκB [54–56] and MAPK [55, 57] signaling increase the expression of the scaffold protein NOD-like receptor pyrin domain-containing 3 (NLRP3). Following a secondary inflammatory stimulus, NLRP3 forms a complex with the apoptosis-associated speck-like protein containing a CARD (ASC) and procaspase 1 and several NLRP3-ASC-procasapse 1 complexes then oligomerize to form the inflammasome [58]. The formation of the inflammasome stimulates the autocleavage and activation of caspase 1, which is critical for the post translational activation of inflammatory cytokines IL1β and IL18 [58].

**Fig. 4:**
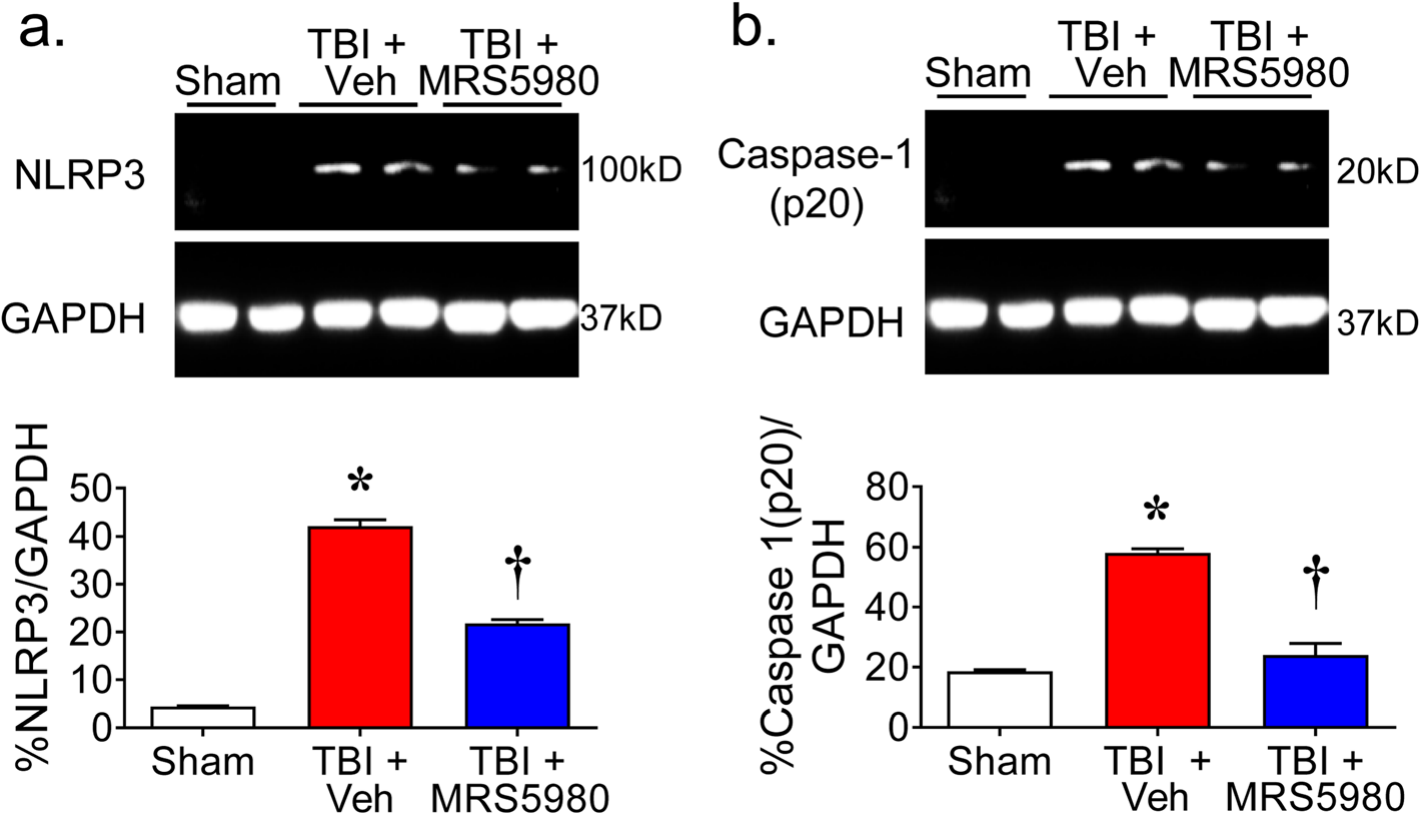
MRS5980 attenuates NLRP3-inflammasome activation following controlled cortical impact TBI. When compared to sham mice, the expression of **a** NLRP3 and **b** cleaved caspase 1 increased in the brain of mice 24 h after controlled cortical impact and vehicle treatment. MRS5980 (1 mg/kg; i.p.) attenuated these changes. The blot in Fig. 3a was probed for NLRP3 and caspase 1 to generate images in **a** and **b** and share the same GAPDH image and data. Uncropped blot images are shown in the supplementary information (Figure S5). Data are mean ± SEM for 5 mice/group and analyzed by two-tailed, one-way ANOVA with Dunnett’s comparisons [**a** *F*(2, 12) = 430.6, *p* = 6.73 × 10^−12^, η^2^ = 0.986; **b** *F*(2, 12) = 79.45, *p* = 1.20 × 10^−7^, η^2^ = 0.930]. *p < 0.05 vs. sham and †*p* < 0.05 vs. TBI + Veh

NLRP3-inflammasome activation occurs within 24 h following TBI [59] and its inhibition reduces the severity of tissue damage following TBI [60–62]. We have recently reported that A_3_AR agonist attenuate the activation of NLRP3 during the neuroinflammatory events in the spinal cord associated with neuropathic pain [63]. In our CCI TBI mouse models, we found increased expression of NLRP3 (Fig. 4a) and cleaved caspase 1 (p20) (Fig. 4b) in the perilesional region of the cortical brain in mice 24 h after injury. This was attenuated in mice with CCI TBI that were treated with MRS5980 (1 mg/kg; i.p.) (Fig. 4).

### MRS5980 attenuates CD4^+^ and CD8^+^ T cell infiltration following CCI

**Fig. 5:**
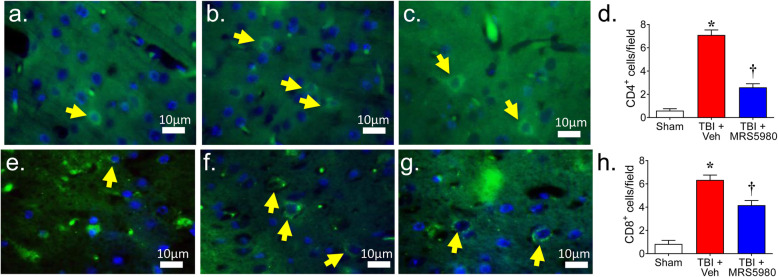
MRS5980 attenuates CD4^+^ and CD8^+^ T cell infiltration following controlled cortical impact TBI. When compared to the number of CD4^+^ (**a**; green) and CD8^+^ (**e**; green) T cells in the brain tissue sham mice, the number of CD4^+^ (**b**) and CD8^+^ (**f**) T cells increased in the brain tissue of mice measured 24 h after CCI and treatment with vehicle. MRS5980 (1 mg/kg; i.p.) attenuated the increase in CD4^+^ (**c**) and CD8^+^ (**g**) T cells in mice with TBI. Quantitation of CD4^+^ (**d**) and CD8^+^ (**h**) T cell infiltration measured from three brain slices for each mouse. Yellow arrows = co-localization between CD4 or CD8 (*green*) and DAPI (*blue*). All images were digitalized at a resolution of 8 bits into an array of 2048 × 2048 pixels. Bar = 20 μm. Data are mean ± SEM for 10 mice/group and analyzed by two-tailed, one-way ANOVA with Bonferroni comparisons [**d:** F(2, 27) = 108.0, *p* = 1.31 × 10^−13^, η^2^ = 0.889 and **h** *F*(2, 15) = 53.14, *p* = 1.56 × 10^−7^, η^2^ = 0.876]. *p < 0.05 vs. sham and †*p* < 0.05 vs. TBI + Veh

The infiltration of CD4^+^ T helper cells have been reported to increase following TBI [36] and contribute to the severity of tissue injury [64, 65]. Consistent with these previous findings, immunofluorescence evaluation of cortical slices from our mice 24 h after CCI revealed increased levels of CD4^+^ and CD8^+^ staining in the perilesional region compared to sham mice (Fig. 5). Administration of MRS5980 (1 mg/kg; i.p.) markedly reduced CD4^+^ and CD8^+^ T cell staining (Fig. 5).

### MRS5980 prevents the development of cognitive impairment after closed head weight-drop TBI

**Fig. 6:**
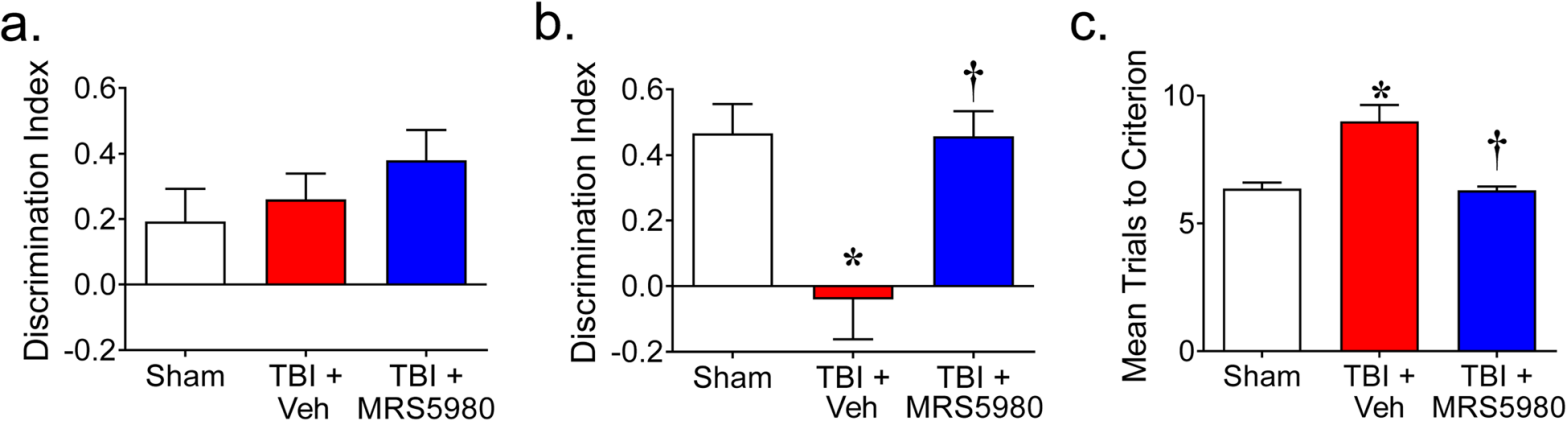
MRS5980 prevents cognitive impairment by 4 weeks after closed head weight-drop TBI. **a** When compared to sham (*n* = 9), there were no differences in the ability of mice 1 week after TBI and vehicle treatment (*n* = 10) to discriminate between conditioned and novel objects or their placement. MRS5890 (1 mg/kg; i.p.; *n* = 10) had no effects on the outcome of NOPRT trials [*F*(2, 26) = 1.092, *p* = 0.351, η^2^ = 0.077]. Animals from the sham (*n* = 2), TBI + Veh (*n* = 1) and TBI + MRS5980 (*n* = 1) groups were excluded because of failure to explore both objects. **b** In contrast, mice with TBI and treated with vehicle (*n* = 9) exhibited increased impairment in recognizing the original object from novel objects or their placement compared to sham (*n* = 9) when tested 4 weeks after TBI. MRS5980 (1 mg/kg; i.p.; *n* = 9) attenuated this impairment in mice with TBI [*F*(2, 26) = 8.976, *p* = 0.00123, η^2^ = 0.428]. Additional animals from the TBI + Veh (*n* = 1) and TBI + MRS5980 (*n* = 1) were excluded because of failure to explore both objects. **c** When compared to sham mice (*n* = 11), the number of trials need to achieve 5 avoidances in 6 consecutive trials in the T maze increased in mice 4–5 weeks after TBI (*n* = 11). Mice with TBI and treated with MRS5980 (1 mg/kg; i.p.; *n* = 10) required fewer T maze trials to achieve learning and memory tasks than untreated mice with TBI [H(2) = 13.58; *p* = 0.00113]. One animal from the TBI + MRS5980 was excluded because it would not perform the task Data are mean ± SD for n mice/group and analyzed by **a**, **b** two-tailed, one-way ANOVA with Bonferroni comparisons or **c** Kruskal-Wallis with Dunn’s comparisons. *p < 0.05 vs. sham and **†***p* < 0.05 vs. TBI + Veh

We next tested whether the beneficial effects of MRS5980 on the underlying TBI pathology and signaling mechanisms translated into protection against cognitive impairment following TBI. Here, we used the closed head weight-drop model of TBI to model the more common closed head TBI seen in the clinic [66]. This model has pathological and neurochemical features similar to those of the CCI model [67, 68]. When memory function was tested 1 week after closed head weight-drop TBI, mice with TBI performed equally well compared to sham mice during NORPT trials (Fig. 6a). However, by 4 weeks, mice with TBI spent significantly less time with the novel objects than the sham mice, indicating a loss of recognition memory of the conditioned object (Fig. 6b). Administration of MRS5980 (1 mg/kg; i.p.) 1 h and every 2 days after trauma attenuated this reduction in recognition memory in mice 4 weeks after TBI (Fig. 6b). MRS5980 had no effect on recognition memory 1 week after TBI (Fig. 6a).

We further explored the effects of MRS5980 on memory and learning using the T maze test. Here, testing 4 weeks after closed head weight-drop TBI revealed that mice with TBI required significantly more trials to achieve 5 avoidances in 6 consecutive trials than sham mice (Fig. 6c). However, those with TBI that were treated with MRS5980 (1 mg/kg; i.p.) 1 h and every 2 days after trauma required fewer trials than mice with TBI (Fig. 6c).

**Fig. 7:**
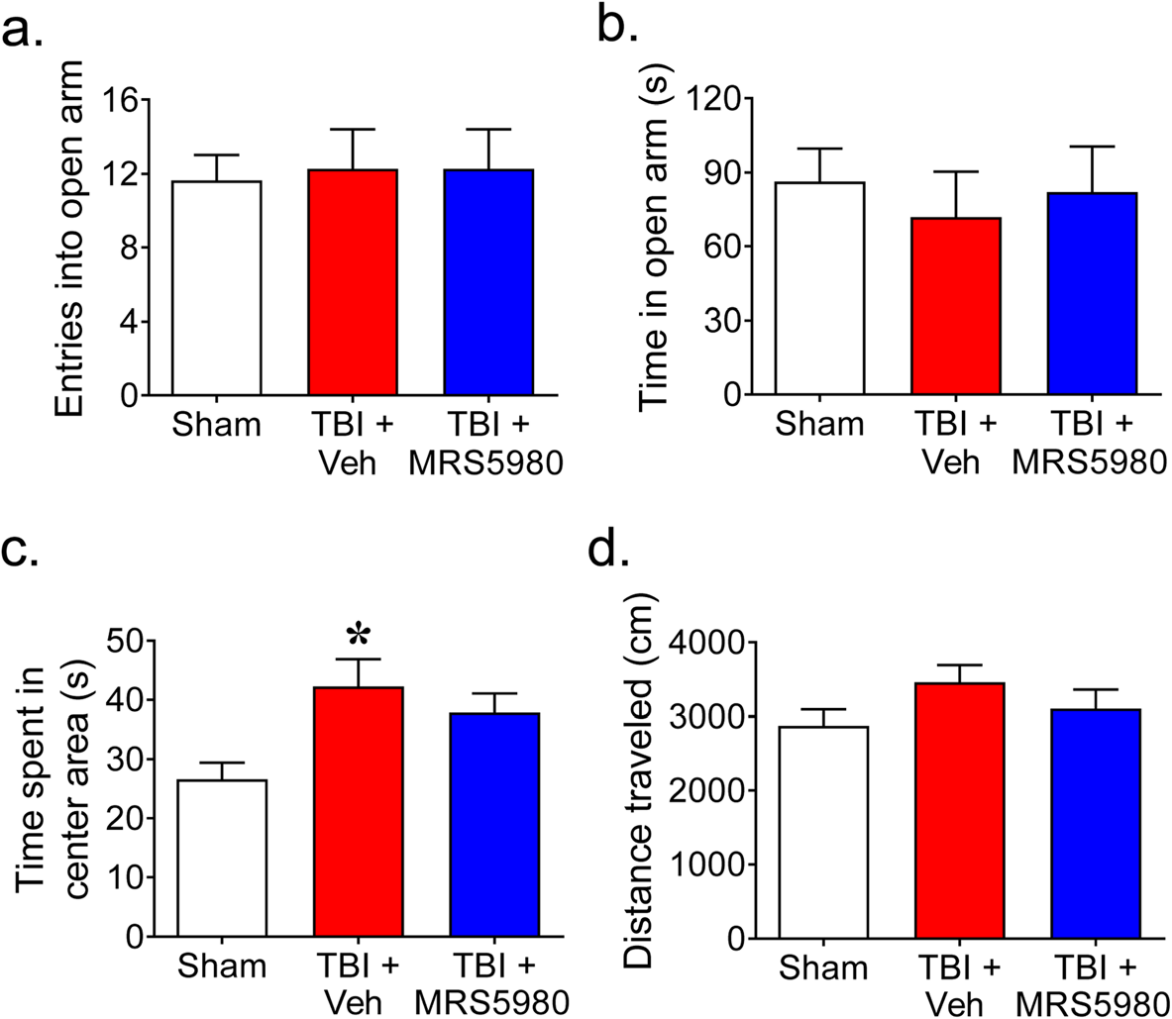
Closed head weight-drop TBI and MRS5980 treatment do not alter mouse activity or cause anxiety. **a**, **b** Elevated plus maze test: when tested 4 weeks after closed head weight drop TBI, there were no differences in the number of entries into **a** or the time spent **b** in the open arms of the elevated plus maze between sham mice (*n* = 11), mice with TBI (*n* = 11) or mice with TBI that were treated with MRS5890 (1 mg/kg; i.p.; *n* = 11). **c**, **d** Open field test: When tested 4 0weeks after closed head weight drop TBI, there was a small but significant increase in time spent in the center of the open area in mice with TBI and treated with vehicle (*n* = 10) compared to sham group (**c**), but not difference in total distance travels (**d**). Mice with TBI that were treated with MRS5890 (1 mg/kg; i.p.; *n* = 11) did not exhibit any differences in time spent in the center (**c**) or distances traveled (**d**) in the open field test compared to the sham group or the mice with TBI treated with vehicle. One animal was excluded from the TBI + Veh group for not moving during testing. Data are mean ± SEM for *n* mice/group and analyzed by **a**, **b** Kruskal-Wallis with Dunn’s comparisons or (**c, d**) two-tailed, one-way ANOVA with Bonferroni comparisons. [**a** H(2) = 0.0368; *p* = 0.982; **b** H(2) = 0.753; *p* = 0.686; **c** *F*(2, 29) = 5.163, *p* = 0.0121, η^2^ = 0.262; **d** *F*(2, 29) = 1.492, *p* = 0.913, η^2^ = 0.0933]. *p < 0.05 vs. sham

Mice with TBI and those treated with MRS5980 (1 mg/kg; i.p.) 1 h and every 2 days after trauma did not exhibit significant differences in the number of entries (Fig. 7a) or time spent on the open arms (Fig. 7b) of an elevated plus maze than sham mice indicating no differences in anxiety. In the open field, mice with TBI and treated with vehicle showed a modest increase in the time spent in the center of an open field than mice in the sham group (Fig. 7c). However, mice with TBI and treated with MRS5980 were not significantly different than sham mice or vehicle-treated mice with TBI (Fig. 7c), indicating that the difference in open field performance was minor and did not impede the ability of the animals to perform other tasks in our behavioral tests. Moreover, there were no significant differences between groups in the total distance traveled in the open field (Fig. 7d). This suggests the behavioral observed changes with TBI or with MRS5980 treatment following TBI were not associated alterations in locomotor activity or increased anxiety.

## Discussion

TBI-induced disability is one of the most pressing health crisis in our society [69, 70]. The Centers for Disease Control and Prevention estimates there are over 2.5 million emergency room visits for TBI each year [66] and an estimated 3–5 million people in the US live with a TBI-related disability [66]. Cognitive impairment is a serious outcome following a TBI [71] that impacts an individual’s quality of life and livelihood [70]. In humans, TBI-related deficits are now thought to be long-term in close to 50% of individuals subjected to a TBI [69]. Impairments several weeks post-TBI can be detected in both mild and severe TBI [72]. Pharmacological interventions for treating TBI are limited.

Our findings now demonstrate that systemic post-TBI administration of MRS5980 protected mice from developing memory impairment as measured by two hippocampal tasks (T-maze and novel object recognition impairment) without altering activity or anxiety levels. Since MRS5980 is > 10,000 times more selective for A_3_AR than for A_1_AR; our findings establish that A_3_AR signaling is beneficial in TBI. The degree and duration of cognitive impairment following TBI is linked to the extent of neurodegeneration caused by the initial insult and secondary injury neuronal death [73]. In an animal stroke model, repeated administration of A_3_AR agonist, IB-MECA, after ischemia was reported to improve cerebral blood flow [74], reduce gliosis and nitric oxide production in the hippocampus and prevent the loss of hippocampal neurons [75]. We now find that the beneficial effects of stimulating A_3_AR signaling on preserving cognitive function following TBI corresponded with mitigation of secondary injury and ischemia in the surrounding brain tissue, supporting the neuroprotective effects of A_3_AR agonists. In our model of CCI TBI, we chose the TTC staining method because it is more rapid than conventional histological assessments and permits a more accurate delineation of injured tissue destined to undergo cell death or degeneration. However, this method is limited in revealing selective neuronal necrosis or other histopathological changes. Future studies investigating the mechanisms engaged by A_3_AR agonists on TBI pathology will have to address how they impact these finer histopathological changes.

Neuronal cell death during the secondary phase of injury is brought on by complex interactions between increased neuroinflammation and oxidative stress, excessive glutamatergic neurotransmission and alterations in blood-brain barrier integrity and cerebral perfusion pressure regulation that lead to edema and ischemia [76]. Tissues damaged during the initial insult release a milieu of signaling molecules, including damage associated molecular patterns (DAMPs) [2, 7] and adenosine triphosphate [7]. DAMPs recognized by toll-like receptors (TLRs) located on glial cells trigger inflammatory cascades result in the production of inflammatory cytokines and chemokines [77] that, in turn, promote oxidative stress [78], enhance neuronal glutamatergic signaling [79] and affect cerebral perfusion pressure [80]. The inflammatory interleukin (IL), IL-1β, is perhaps the most studied cytokine in TBI and found to be elevated with the first hours following TBI [2, 81]. IL-1β transcription is induced by DAMP-stimulation of toll-like receptors in microglia during TBI and requires post-translation processing by caspase 1 activated by inflammasomes, such as NLRP3 [58]. The levels of NLRP3 inflammasome components and IL-1β are elevated in the cerebrospinal fluid of TBI patients [82]. Moreover, adenosine triphosphate levels rise following TBI [83] and can act as the secondary trigger for inflammasome oligomerization by binding the adenosine triphosphate-gated P2X receptor cation channel subtype 7 (P2X7) [58, 83]. Inhibition of P2X7 following TBI prevents IL-1β expression, glial activation and neuronal cell death [84]. The effects of IL-1β in brain injury have also been linked to promotion granulocytosis that can drive neuronal cell death [83], induction glutamatergic neurotoxicity [85], and reduction in cerebral blood flow to exacerbate brain infarction [80]. The elimination of IL-1β signaling in IL-1 receptor (*IL1r*) knockout mice has been shown to significantly improve cognitive function following TBI ([81]. Here, in our CCI model, we found systemic administration of MRS5980 attenuated NLRP3 expression, as well as the NFκB and MAPK regulatory pathways, and caspase 1 activation suggesting that the beneficial effects of A_3_AR activation on cognitive function and tissue injury is related to the prevention of adverse NLRP3-IL-1β signaling. These actions are consistent with our previously reported finding of the effects of A_3_AR in the CNS in neuropathic pain models where we found A_3_AR agonists blocked NLRP3 expression and activation [63] and reduced IL-1β expression in the spinal cord [63, 86]. In our neuropathic pain models, A_3_AR mitigation of NLRP3-dependent IL-1β in spinal cord was associated with increased IL-10 [63, 86]. In TBI models, IL-10 not only attenuates neuroinflammation by reducing inflammatory cytokine production, but also was found be neuroprotective by increasing cerebral blood flow and reducing brain infarction [87].

Although A_1_AR and A_2A_AR expression is greater than A_3_AR in the brain [11, 21, 25], the rapid rise in extracellular adenosine shortly following TBI is likely to engage A_1_AR, A_2_AAR, and A_3_AR to provide acute modulation of the neuroinflammation events provoked by the primary tissue injury. However, these levels appear to be transient with only a short burst of adenosine release when cerebral blood flow does not match the oxygen consumption within the site of injury. The duration of these episodic adenosine releases may be shortened by derangements in the metabolism of extracellular adenosine following TBI [88]. Regulation of extracellular adenosine is largely driven by the intracellular adenosine kinase (ADK), which converts intracellular adenosine to adenosine monophosphate [89]. Increased adenosine kinase activity, such as that seen under pathological conditions, depletes intracellular adenosine and draws extracellular adenosine down its gradient through passive channels [89]. Increased expression of ADK in astrocytes following penetrative (blade-induced) TBI has been found associated with the development of astrogliosis-associated neuronal death; knocking down ADK in astrocytes reduced their proinflammatory phenotype [90]. Moreover, recent studies show that neural stem cell proliferation following controlled cortical impact TBI was impaired in mice overexpressing ADK; whereas its pharmacological inhibition promoted neural stem cell proliferation following TBI [91]. Extracellular adenosine levels are further influenced by the conversion of extracellular ATP to adenosine by ectonucleotidases [92, 93]. In addition to increased adenosine release after TBI, there is release of ATP from the primary injury site that triggers neuroinflammatory responses [83], such as activating microglia [94] and triggering P2X7R-mediated inflammation [84]. However, in penetrative (cortical stab) TBI models, adenosine monophosphate hydrolysis (CD73 activity) was found impaired in cortical regions [95, 96] and the hippocampus [96]. Collectively, the events would lead to short durations of released adenosine and low adenosine level between these episodes, which at its best may only be sufficient to engage A_1_AR and A_2A_AR, thus creating deficiencies in long-term beneficial endogenous A_3_AR signaling and favor ongoing neuroinflammatory signaling.

## Conclusions

Our findings using highly selective A_3_AR agonists demonstrate that activation of the A_3_AR following TBI protects against tissue damage, brain infarct, neural inflammation and cognitive dysfunction. Given the good safety profiles of picladenosin and namodenosin, A_3_AR presents an exciting potential therapeutic target for prevention of permanent damage due to TBI.

## Supplementary Information

**Additional file 1: Figure S1.** IκBα and GAPDH Western blots. Uncropped images of Western blot images shown in Fig. 3a. *Yellow box* demarcates the cropped area. **Figure S2.** NFBα and GAPDH Western blots. Uncropped images of Western blot images shown in Fig. 3b. *Yellow box* demarcates the cropped area. **Figure S3.** Phosphorylated and total p38 Western blots. Uncropped images of Western blot images shown in Fig. 3c. *Yellow box* demarcates the cropped area. **Figure S4.** Phosphorylated and total ERK Western blots. Uncropped images of Western blot images shown in Fig. 3d. *Yellow box* demarcates the cropped area. **Figure S5.** NLRP3, caspase 1 and GAPDH Western blots. Uncropped images of Westernblot images shown in Fig. 4. *Yellow box* demarcates the cropped area. Blots shown in Fig. 3a and S1 were probed for NLRP3 and caspase 1 and share the same GAPDH image.

## Data Availability

The datasets used and/or analyzed during the current study are available from the corresponding author on reasonable request.

## Abbreviations

A_1_AR: Adenosine receptor subtype 1
A_2A_AR: Adenosine receptor subtype 2A
A_3_AR: Adenosine receptor subtype 3
ASC: Apoptosis-associated speck-like protein containing a caspase activation and recruitment domain
CARD: Caspase activation and recruitment domain
CCI: Controlled cortical impact
Cl-IB-MECA: 1-[2-Chloro-6-[[(3-iodophenyl)methyl]amino]-9H-purin-9-yl]-1-deoxy-N-methyl-β-D-ribofuranuronamide
DAMPs: Danger associated molecular patterns
DMSO: Dimethyl sulfoxide
ERK: Extracellular signal-regulated kinase
GAPDH: Glyceraldehyde 3-phosphate dehydrogenase
GPCR: G protein-coupled receptor
IB-MECA: 1-Deoxy-1-[6-[((3-Iodophenyl)methyl)amino]-9H-purin-9-yl]-N-methyl-β-D-ribofuranuronamide, N6-(3-Iodobenzyl)adenosine-5′-N-methyluronamide
IκBα: Nuclear factor of kappa light polypeptide gene enhancer in B cells inhibitor, alpha
IL: Interleukin
i.p.: Intraperitoneal
MAPK: Mitogen-activated protein kinase
MRS5980: (1*S*,2*R*,3*S*,4*R*,5*S*)-4-(2-((5-chlorothiophen-2-yl)ethynyl)-6-(methylamino)-9*H*-purin-9-yl)-2,3-dihydroxy-*N*-methylbicyclo[3.1.0]hexane-1-carboxamide
NFκB: Nuclear factor of kappa light polypeptide gene enhancer in B cells
NLRP3: NOD-like receptor pyrin domain-containing 3
NOD: Nucleotide-binding oligomerization domain-containing protein
NOPRT: Novel object-placement recognition test
P2X7: Adenosine triphosphate-gated P2X receptor cation channel subtype 7
TBI: Traumatic brain injury
v/v: Volume-to-volume
w/v: Weight-to-volume
