# Supplementary material for: Adenosine A3 receptor as a novel therapeutic target to reduce secondary events and improve neurocognitive functions following traumatic brain injury
Source: J Neuroinflammation. 2020 Nov 12;17:339. doi: 10.1186/s12974-020-02009-7 (PMC7659122; doi:10.1186/s12974-020-02009-7)
Supplement: Supplementary file 1 — Additional file 1: Figure S1. IκBα and GAPDH Western blots. Uncropped images of Western blot images shown in Fig. 3a. Yellow box demarcates the cropped area. Figure S2. NFBα and GAPDH Western blots. Uncropped images of Western blot images shown in Fig. 3b. Yellow box demarcates the cropped area. Figure S3. Phosphorylated and total p38 Western blots. Uncropped images of Western blot images shown in Fig. 3c. Yellow box demarcates the cropped area. Figure S4. Phosphorylated and total ERK Western blots. Uncropped images of Western blot images shown in Fig. 3d. Yellow box demarcates the cropped area. Figure S5. NLRP3, caspase 1 and GAPDH Western blots. Uncropped images of Westernblot images shown in Fig. 4. Yellow box demarcates the cropped area. Blots shown in Fig. 3a and S1 were probed for NLRP3 and caspase 1 and share the same GAPDH image. [file 12974_2020_2009_MOESM1_ESM.docx]

Supplementary information for:

**Adenosine A_3_ receptor as a novel therapeutic target to reduce secondary events and improve neurocognitive functions following traumatic brain injury.**

Susan A. Farr^1,2,3,4^, Salvatore Cuzzocrea^5^, Emanuela Esposito^5^, Michela Campolo^5^, Michael L. Niehoff^2^, Timothy M. Doyle^3,4^ and Daniela Salvemini^3,4*^.

^1^Veterans Affairs Medical Center 915 N Grand Blvd, St. Louis, MO 63106

^2^Department of Internal Medicine, Division of Geriatric Medicine, Saint Louis University School of Medicine, 1402 S. Grand Blvd, St. Louis MO, USA 63104

^3^Department of Pharmacology and Physiology, Saint Louis University School of Medicine, 1402 S. Grand Blvd, St. Louis MO, USA 63104

^4^Henry and Amelia Nasrallah Center for Neuroscience, Saint Louis University School of Medicine, 1402 S. Grand Blvd, St. Louis MO, USA 63104

^5^Department of Clinical and Experimental Medicine and Pharmacology, University of Messina, Messina 98122 Italy.

***Corresponding author:** E-mail address: daniela.salvemini@health.slu.edu. ORCID ID: 0000-0002-0612-4448. Address: 1402 South Grand Blvd, St. Louis, MO 63104, USA, Phone: 1-314-977-6430, Fax: 1-314-977-6411


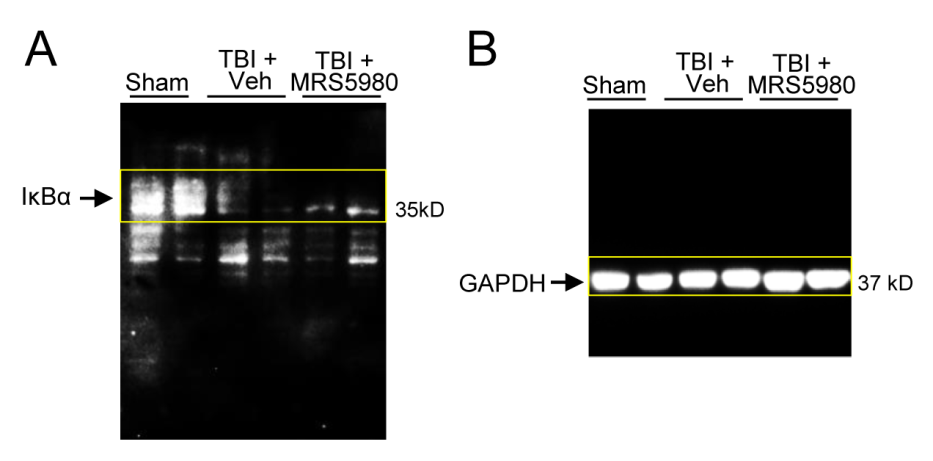


**Figure S1. IκBα and GAPDH Western blots.** Uncropped images of Western blot images shown in **Fig. 3a**. *Yellow box* demarcates the cropped area.

**
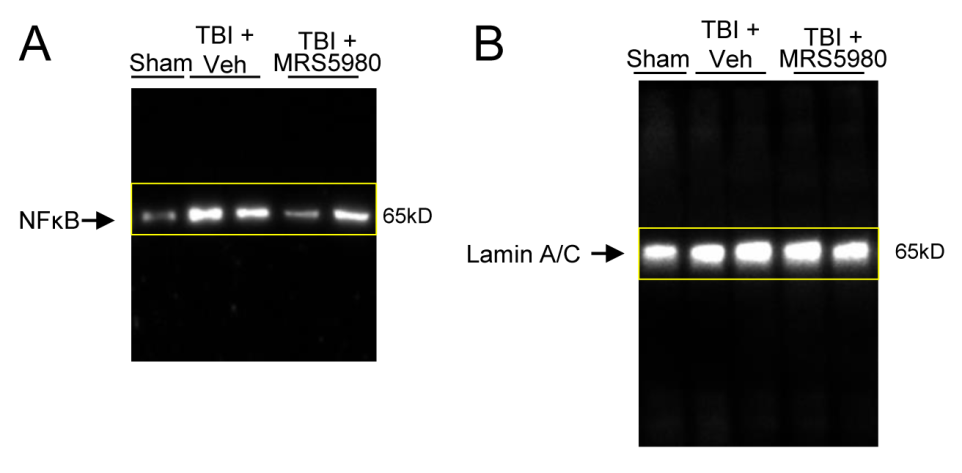
**

**Figure S2. NFBα and GAPDH Western blots.** Uncropped images of Western blot images shown in **Fig. 3b**. *Yellow box* demarcates the cropped area.


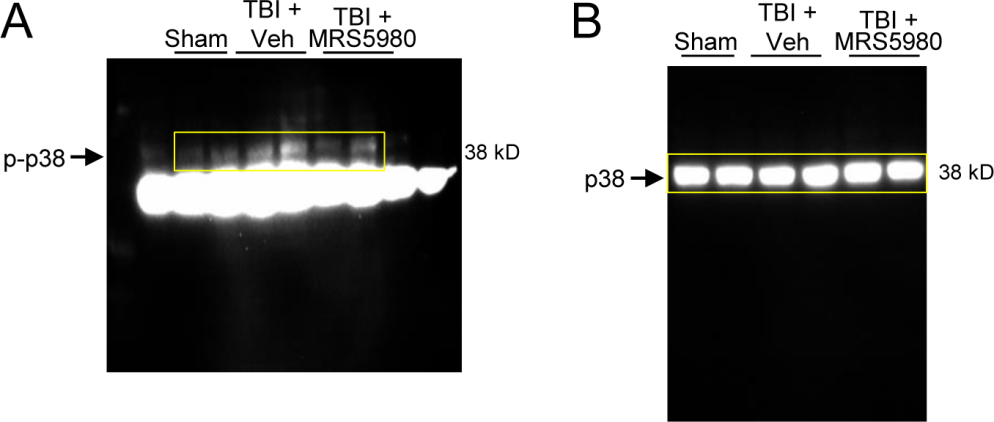


**Figure S3. Phosphorylated and total p38 Western blots.** Uncropped images of Western blot images shown in **Fig. 3c**. *Yellow box* demarcates the cropped area.


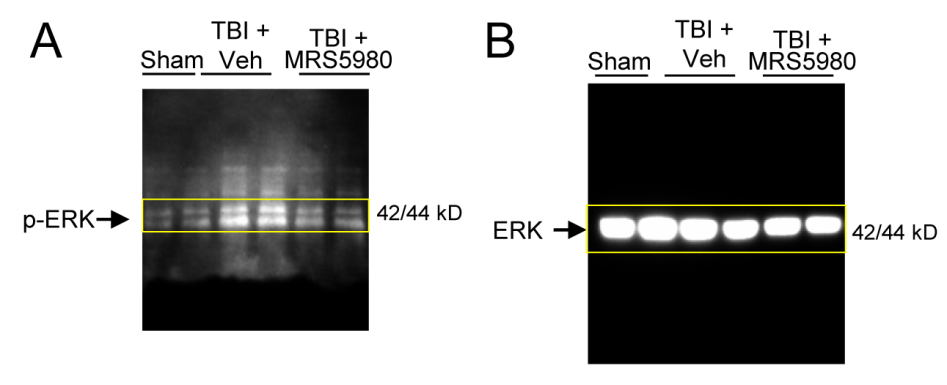


**Figure S4. Phosphorylated and total ERK Western blots.** Uncropped images of Western blot images shown in **Fig. 3d**. *Yellow box* demarcates the cropped area.


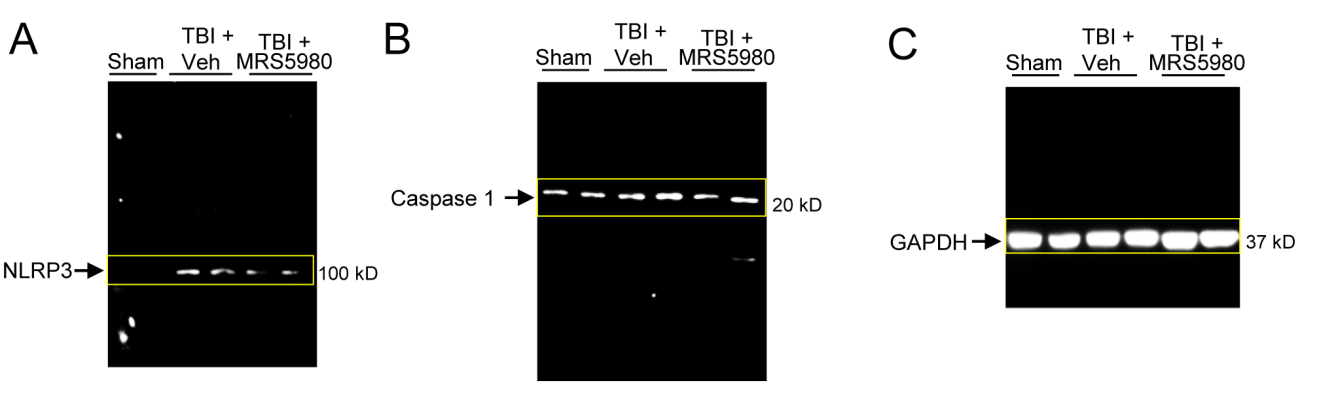
**Figure S5. NLRP3, caspase 1 and GAPDH Western blots.** Uncropped images of Westernblot images shown in **Fig. 4**. *Yellow box* demarcates the cropped area. Blots shown in **Figs. 3a and S1** were probed for NLRP3 and caspase 1 and share the same GAPDH image.
